# Towards a qAOP framework for predictive toxicology - Linking data to decisions

**DOI:** 10.1016/j.comtox.2021.100195

**Published:** 2022-02

**Authors:** Alicia Paini, Ivana Campia, Mark T.D. Cronin, David Asturiol, Lidia Ceriani, Thomas E. Exner, Wang Gao, Caroline Gomes, Johannes Kruisselbrink, Marvin Martens, M.E. Bette Meek, David Pamies, Julia Pletz, Stefan Scholz, Andreas Schüttler, Nicoleta Spînu, Daniel L. Villeneuve, Clemens Wittwehr, Andrew Worth, Mirjam Luijten

**Affiliations:** a European Commission, Joint Research Centre (JRC), Ispra, Italy; b Liverpool John Moores University, Liverpool, United Kingdom; c Humane Society International, Brussels, Belgium; d Edelweiss Connect GmbH, Technology Park Basel, Basel, Switzerland; e Institut National de l'Environnement Industriel et des Risques (INERIS), Verneuil-en-Halatte, France; f BASF, Ludwigshafen, Germany; g Wageningen University & Research, Wageningen, The Netherlands; h Maastricht University, Maastricht, The Netherlands; i University of Ottawa, Ottawa, Canada; j Department of Physiology, Lausanne and Swiss Centre for Applied Human Toxicology (SCAHT), University of Lausanne, Lausanne, Switzerland; k Helmholtz Centre for Environmental Research GmbH – UFZ, Leipzig, Germany; l US Environmental Protection Agency, Great Lakes Toxicology and Ecology Division, Duluth, MN, USA; m National Institute for Public Health and the Environment (RIVM), Bilthoven, The Netherlands

**Keywords:** quantitative Adverse Outcome Pathway (qAOP), Hazard assessment, Weight of evidence (WoE), *In vitro* data, *In silico* data, Predictive toxicology

## Abstract

The adverse outcome pathway (AOP) is a conceptual construct that facilitates organisation and interpretation of mechanistic data representing multiple biological levels and deriving from a range of methodological approaches including *in silico*, *in vitro* and *in vivo* assays. AOPs are playing an increasingly important role in the chemical safety assessment paradigm and quantification of AOPs is an important step towards a more reliable prediction of chemically induced adverse effects. Modelling methodologies require the identification, extraction and use of reliable data and information to support the inclusion of quantitative considerations in AOP development. An extensive and growing range of digital resources are available to support the modelling of quantitative AOPs, providing a wide range of information, but also requiring guidance for their practical application. A framework for qAOP development is proposed based on feedback from a group of experts and three qAOP case studies. The proposed framework provides a harmonised approach for both regulators and scientists working in this area.

## Highlights

• Chemical toxicity assessment depends on the quantification of kinetics and dynamics.
• Quantitative AOPs (qAOPs) are toxicodynamic models based on Adverse Outcome Pathways.
• Existing e-resources could form the basis of an e-infrastructure for qAOP modelling.
• Best practices for qAOP development, assessment and application are needed.
• Three qAOP case studies are presented to illustrate a modelling workflow.

## Introduction

The Adverse Outcome Pathway (AOP) concept was proposed in 2010 as an organisational framework to facilitate integration and interpretation of mechanistic toxicity data, to be included as an important component of a more predictive chemical safety assessment paradigm [1], [2], [3] and as a flexible and practical tool supporting 21st century toxicology [4]. An AOP is a chemical-agnostic theoretical construct, representing a sequence of measurable “events”. These events describe the progression from the initial perturbation of a biological system by a stressor (a molecular initiating event [MIE]) to an eventual adverse effect on the health or survival of an organism or population (an adverse outcome [AO]). AOPs are developed and evaluated based on the consideration of multiple lines of evidence in a weight of evidence (WoE) approach, centred on establishing a causal connection between measurable biological events [5], [6], [7], [8], [9]. AOPs describe the key events (KEs) and key event relationships (KERs) from the initial interaction of a stressor with its biological target(s), through the intermediary steps in the biological pathway, to a final AO. KERs serve as a type of biological “if, then” statement. If event A is observed at sufficient magnitude, *then* event B can be expected to occur. The support for the “if, then” statement is provided in terms of the biological plausibility and patterns of empirical support for the relationship. The quantitative understanding of the KERs should define the specific conditions under which event A will cause event B, and the magnitude or probability of change in event B as a function of event A. Several examples of AOPs developed for different adverse effects (such as cancer, damage to specific organs, immune responses and many others) in humans and environmental organisms are available in the AOP wiki (aopwiki.org) [10], [11], [12].

While the AOP framework provides a systematic approach for qualitatively organising knowledge, mathematical models of the key event relationships (at various scales) provide the means and platform to quantitatively integrate current biological understanding to facilitate interpretation and extrapolation [13] and address data gaps. Mathematical models enable quantitative prediction of KERs and AOs taking into account available biological data and their relationships. Conolly et al. [14] defined a quantitative Adverse Outcome Pathway (qAOP) as an *AOP for which the quantitative understanding of the relationship that underlie transitions from one KE to the next, and critical factors that modulate those relationships, are sufficiently well defined to allow quantitative prediction of the probability or severity of the AO for a given level of perturbation of the MIE.* Where this level of understanding can be achieved, AOPs can be used not only to support hazard identification and characterisation, but also to evaluate risk when posed with an appropriate exposure scenario and complementary information on the biokinetic properties of a stressor. Quantification is necessary for a more reliable prediction of stressor (including chemical) specific effects, including potency, which is a prerequisite for risk assessment.

Predictions from models that incorporate information on complex biological relationships, may have greater biological fidelity to support hazard and risk assessment than models with simplified assumptions [14]. Perkins et al. [15] described qAOPs as mathematical constructs that model the response-response relationships of KERs in an AOP. Quantitative AOP models can incorporate complex biological mechanisms, such as feedback loops, thresholds, and signalling cascades that are generally embedded in the KE or KER of descriptive AOPs. To be useful in a quantitative context, the factors impacting relationships between KEs must be sufficiently understood. AOP modelling methodologies range from statistical/machine learning approaches to mechanistic approaches using ordinary differential equations to individual-based models, and should be chosen according to the questions being asked and the data available [15], [16]. Thus, there is a need to identify, extract and utilise reliable sources of data and information to inform these quantitative considerations in AOP development.

An extensive and growing range of electronic, digital resources (hereafter named e-resources) are available to support the modelling of qAOPs [17], [18]. Digital resources can be defined as web tools/interfaces, datasets/databases, mathematical models that have been conceived and created digitally or by converting chemical and biological data and information to a digital format. These should follow the principles of findability, accessibility, interoperability, and reusability (FAIR data principles) [19].

E-resources capture data related to the toxicological effects of chemicals at a range of levels of biological organisation and provide access to a broad range of predictive software tools (e.g., QSAR tools), to support predictive chemical risk assessment. However, these resources build from diverse heterogenous sources and are not tailored specifically to qAOP development. Consequently, there is a demand for a mapping exercise as well as better description of the resources to develop qAOPs that could facilitate their interoperability. In addition, it is known that, “*a good experiment reported badly is worthless*”, therefore the lack of Good *In Vitro* Reporting Standards (GIVReSt) can make the use and interoperability of generated data occasionally difficult [20], [21]. The Guidance Document on Good *In Vitro* Method Practices (GIVIMP), published by Organisation for Economic Co-operation and Development (OECD) in 2018, is a first step to generate quality data, this guidance provides direction for the development and implementation of *in vitro* methods and tackles key elements to ensure resulting data are reproducible and well- reported [22]. The role of e-resources and the capability to enhance the development of quantitative AOPs is currently underutilised. These resources might enrich current and future AOP development. To this end a workshop entitled “e-Resources to Revolutionise Toxicology: Linking Data to Decisions”, was held at the Lorentz Center (Leiden, The Netherlands) in October 2019 with EURL ECVAM, the Dutch National Institute for Public Health and the Environment (RIVM, NL) and Liverpool John Moores University (LJMU, UK) as co-organisers. The main objectives of this workshop were:

1. to map e-resources and identify how they could enhance the AOP framework;
2. to design and establish a workflow for quantitative AOP development based on available data and e-resources.

This paper highlights the main outcomes of this workshop including a general workflow to guide the quantification of an AOP, enabling the development of qAOP models.

## Key learnings when exploring qAOP development for three different adverse outcomes

During the one-week workshop, three case studies were elaborated covering different AOs, namely skin sensitisation, neurotoxicity and carcinogenicity. The AOPs were used to analyse and model selected KERs of the three different AOs. First, the participants mapped out available resources (datasets, modelling methodologies) to build quantitative KERs between KEs, including their typical applications and data requirements (a full list of these resources is available in the Supplementary Material). Additional discussions focused on which considerations should be taken into account for assessment of confidence (i.e. weight of evidence) in the supporting data and its relationship with quantification. With regard to potential regulatory applications, the role of transparent documentation of the proposed approach was also discussed. As such, these case studies provided a proof-of-principle to address critical questions in the development and use of AOP-informed mathematical/computational models, or qAOP models. In Table 1 we summarise the main lessons learned when elaborating these three case studies, including: *i)* AOP description; *ii)* data collection, curation and harmonisation (datasets and e-resources); and *iii)* modelling approaches.

**Table 1:** Summary of key learnings from the three case studies.

			**Key learnings**			
**Adverse outcome**	**AOPs or AOP networks**	**Reference**	***AOP description***	***Datasets***	***e-resources***	***Modelling approach***
Skin sensitisation	“Covalent Protein binding leading to Skin Sensitisation” – AOP 40	https://aopwiki.org/aops/40	• Linear AOP • OECD endorsed • Good level of documentation for all KEs and KERs	• Data-rich AOP: several datasets available in literature e.g. dataset by Urbisch et al. [24] and evaluated by Hoffmann et al. [25] • Data available for *in vitro*, *in chemico* and *in vivo* studies. Full matrix for ≥ 120 chemicals. Human data also available for most of the chemicals • Diversity of measurement units between sources (e.g. % of effect at a fixed concentration and time exposure vs. concentration at which effect is 3-fold). Need to normalise data points.	OECD QSAR toolbox [33]	Bayesian networks that allow combination of diverse datasets
(Developmental) neurotoxicity	AOPs for neurotoxicity - AOP 3, 12, 13, 17, 42, 48, 54, 134, 260	[17]AOP-Wiki (https://aopwiki.org/) for the single AOPs	• Network of AOPs • AOPs at different level of development (from under development to OECD endorsed) • Focus on intermediate and interconnected KEs and KERs	• Data limitations: availability of precompiled datasets. Literature reviews needed to extract quantitative relevant information about KEs from literature • Data available from *in vitro* and *in vivo* studies • Need to convert different data points to the same unit of measurements, apply the same weight to individual studies or data points in a single study	Developmental NeuroToxicity Data Integration and Visualization Enabling Resource (DNT-DIVER) [34]	Bayesian networks that can be used with relatively sparse data or when multiple pathways can affect the AO
Carcinogenicity	“Cyp2E1 Activation Leading to Liver Cancer” – AOP 220	https://aopwiki.org/aops/220	• Linear AOP • Under final stage of OECD review • Focus on late KEs and KERs predictive of AO	• Good data availability: datasets available from literature and regulatory dossiers • Available data mainly originated from *in vivo* studies • Importance of well- designed studies of dose–response relationships for KEs at several levels of biological organisation at relevant time points	OECD eChemPortal [35]EFSA OpenFoodTox database [36]PubMed (keywords used: substance name, CAS N., proliferation, tumour) [37]EFSA publications (e.g. EFSA’s Peer Review Conclusions on Pesticides) [38]Health Canada’s Assessment Reports [39]	An equation (similar to Zgheib et al. [31]) describing dose response relationship was applied and data were also normalised using PBK modelling

### Case study 1: Skin sensitisation

#### AOP description

**Fig. 1:**
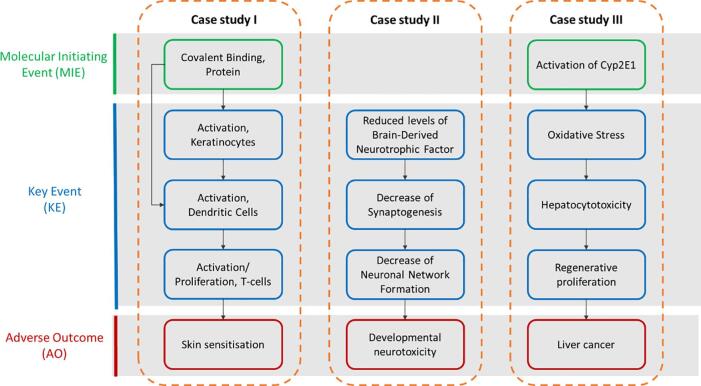
Schematic description of the AOPs selected for the three case studies. Case Study I: AOP 40, “Covalent Protein binding leading to Skin Sensitisation”, covering the skin sensitisation endpoint. Case study II: set of KEs derived from network of (developmental) neurotoxicity AOPs (see Fig. 2). Case study III: AOP 220, “Cyp2E1 Activation Leading to Liver Cancer” as example of a pathway underlying carcinogenicity. Boxes circled in green indicate molecular initiating events (MIEs), in blue key events (KEs) and in red adverse outcome (AO). (For interpretation of the references to colour in this figure legend, the reader is referred to the web version of this article.)

The AOP “Covalent Protein binding leading to Skin Sensitisation” (AOP40, aopwiki.org) is well studied and documented and has been endorsed by the OECD [23]. AOP 40 is built on four KEs, where the molecular initiating event (MIE or KE1) is the covalent binding to skin proteins (termed haptenation), by electrophilic stressors. KE2 is the activation of epidermal keratinocytes, and the activation (maturation) and mobilisation of Langerhans and dermal dendritic cells (DC) is KE3. KE4 corresponds to the activation/proliferation of antigen-specific naïve T cells by DC-mediated antigen presentation (KE4) (Fig. 1). Thus, the well-defined linear nature and status of the KEs makes it a useful target to attempt quantification.

#### Datasets & e-resources

In this case study the wealth of freely available data related to skin sensitisation was considered. They span human data, *in vivo* data from regulatory studies as well as data from *in chemico* and *in vitro* alternative methods. Several datasets with skin sensitisation data from human, local lymph node assay (LLNA), and individual methods representing KEs are available in the literature. To investigate the development of the qAOP, the dataset published by Urbisch et al. [24] and evaluated by Hoffmann et al. [25] was investigated. This was the largest dataset available presenting a full data matrix across the AOP including information for more than 120 chemicals, i.e. human data characterising KE1, KE2, and KE3, and LLNA data to investigate the AO. Studies on skin sensitisation endpoints for several chemicals are also available in curated e-resources such as OECD QSAR Toolbox (links in Table 1).

Several limitations in the data for skin sensitisation to support qAOP development were highlighted. The first limitation was the lack of dose response information for each of the KEs measured in either *in vivo* or *in vitro.* The second limitation was the lack of comparability of reported results for each KE between different studies. These may vary from a concentration causing a specific effect (e.g. the LLNA endpoint: concentration at which the induced proliferative responses in draining lymph nodes is three-fold (EC3)) to a percentage effect (e.g. direct peptide reactivity assay (DPRA): percentage of peptide depletion). Thus, even where dose–response information is available for multiple KEs, those data are often not reported in units that are easily interconverted and compared. Thus, from the outset, effort is required to standardise and scale data and responses from various assays for use in qAOP development.

#### Modelling approaches

Among the several modelling methods considered to model the qAOP, the Bayesian approach was favoured as it allows the combination of diverse datasets, provides confidence scores of the predictions, and can assess the added value of including additional measured data. In addition, the structure of the Bayesian network itself may provide an answer to whether all assays are required to predict the AO, or only a small number of them.

### Case study 2: (developmental) neurotoxicity

#### AOP description

**Fig. 2:**
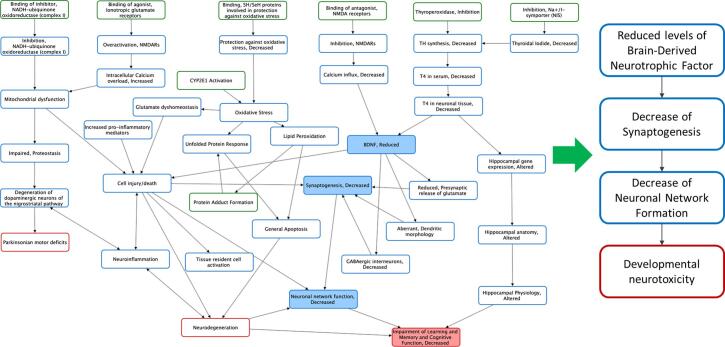
Case Study II. AOP network covering (AOP 3, 12, 13, 17, 42, 48, 54, 134, 260) developmental neurotoxicity (based on Spînu et al. [17]) was used to derived a linear chain of KEs (see right side and Fig. 1).

The second case study addressed an AOP network for developmental neurotoxicity described by Spinu et al. [17]. Briefly, the network connects nine AOPs (AOP 3, 12, 13, 17, 42, 48, 54, 134, 260 available in the AOP-Wiki https://aopwiki.org) by means of shared KEs (Fig. 1, Fig. 2). The status of these AOPs within the OECD programme ranges from “under development” to “TFHA/WNT endorsed”^3^. The AOPs within the network contain adjacent KERs and share three neurotoxicity-related AOs defined as neurodegeneration, Parkinsonian motor deficits and impairment of learning and memory/decrease or cognitive function [17].

KERs which connect KEs centrally located in the network and representing nodes of convergence of many AOPs, were prioritised for quantification. The KE “cell injury/death” represented the most highly connected node/intermediate KE in the network. However, it was considered too non-specific with respect to the (developmental) neurotoxicity network to facilitate collection of data relevant for development of a quantitative model. Therefore, reduction of Brain-Derived Neurotrophic Factor (BDNF) leading to the subsequent decrease of synaptogenesis and decrease in neural network function were selected as set of KEs suitable for KER quantification (Fig. 2). The choice of common KEs for modelling represents a pragmatic decision, based not only on the topological analytics indicating the most centrally connected common KEs, but also on the availability of existing data and cost-effectiveness of obtaining new experimental data. For example, the cost of performing imaging experiments on cell cultures (synaptogenesis) may be different than the cost of electrophysiological (microelectrode array) studies (neural network activity). Irrespective of the choice of common KEs, a residual uncertainty concerns the potential impact of excluded pathways. Further details of this qAOP and the underlying modelling approach are provided in Spînu et al (submitted) and [26].

#### Datasets & e-resources

In this case study, it was noted that an extensive data collection exercise would be required due to the limited availability of precompiled data. Quantitative data extracted from literature needed to be compiled, annotated and organised in a structured and appropriate format to ensure compatibility with a suitable modelling technique. The Developmental NeuroToxicity Data Integration and Visualization Enabling Resource (DNT-DIVER) by National Toxicology Program provides a web-based curated collection of datasets from multiple DNT assays (link in Table 1). In this e-resource, benchmark concentration analysis was applied to compare data obtained in assays using different species (e.g. human-based cell assays or animal models such as zebrafish and planaria) and addressing various endpoints (e.g. neuron outgrowth, protein accumulation and behaviour in fish) [27]. The assessment of variability between different assays was identified as a major obstacle. The same endpoints can be investigated in different test systems (e.g. animal models or human samples) or by using different experimental techniques. For example, synaptogenesis can be assessed by using imaging techniques or measuring protein content. Therefore, it is useful to have access to the experimental protocols to understand how data were generated. Moreover, as part of the data processing, data points have to be converted to the same unit of measurement (e.g. BDNF concentration as % of control). Another important consideration is the weight to be assigned to individual studies or data points in a single study, for example, where multiple concentrations are tested in different test systems. The inclusion of concordance tables in the AOP-Wiki [28] would support the development of harmonised and structured datasets for quantitative modelling. Datasets could be extended over time to include additional data. Ideally, quantitative datasets would be compiled in a machine-readable format to support their reusability and comply with FAIR data Principles.

#### Modelling approaches

As for Case study 1, the Bayesian network was identified as the most suitable approach for quantitative modelling of literature data of neurotoxicity-related AOPs. Moreover, Bayesian networks allow complex scenarios to be modelled by a relatively simple approach with little requirement for parameterisation. They can be built on expert knowledge as well as training data sets. In particular, they can be used with relatively sparse quantitative data and when multiple pathways can affect the AO. BNs can be tested for different network architectures and also for various combinations of events.

### Case study 3: Carcinogenicity

#### AOP description

The third case study focused on carcinogenicity and the AOP on “Cyp2E1 Activation Leading to Liver Cancer” (AOP 220 in the AOP-Wiki) (Fig. 1). At the time of writing, this AOP is in the final stage of OECD review and approval. AOP 220 presents the chronic CYP2E1 activation (MIE) leading to liver cancer, which induces a series of cascade events. Oxidative stress (KE1) caused by CYP2E1 activation can induce hepatotoxicity (KE2), leading to liver cancer. The liver has a regenerative capacity through cellular proliferation (KE3) that is protective against injury. However, uncontrolled cellular proliferation resulting from chronic CYP2E 1 activation promotes hepatocarcinogenesis (AO) [29] through fixation of spontaneous mutations. In this case study, the late KERs linking upstream cellular toxicity leading to downstream regenerative cellular proliferation leading to the AO (liver cancer) were selected as starting point for quantification. Selection of these KERs from AOP 220 was based on the extent of data available as basis for quantification and on consideration that proximity to the AO to facilitate predictive application in hazard characterisation for regulatory purposes.

#### Datasets & e-resources

The first step in data collection was to identify (chemical) stressors that are relevant for the AOP of interest. For the selected KEs and KERs of AOP 220, most of the quantitative data were retrieved from chemical assessment reports from regulatory agencies, which included peer-review of relevant toxicological studies. Alternatively, data could be derived directly from the scientific literature, provided that results are sufficiently documented, and the study is adequately designed (preferably conducted according to relevant OECD Test Guidelines). Robust tumour incidence data were relatively easily available, while data on histopathological measurements of cell toxicity and data on regenerative proliferation, at relevant time periods were more difficult to identify. This was partly due to the fact that regenerative proliferation is not a requirement in the cancer bioassay [30]. Briefly, quantitative data for a list of chemical stressors, associated with a possible cancer adverse outcome, were collected from several tools and databases, including some of the e-resources identified in the workshop, such as OECD’s eChemPortal and EFSA’s OpenFoodTox database (links in Table 1). In addition, PubMed, EFSA’s Peer Review Conclusions on Pesticides and Health Canada’s Assessment Reports, were also taken into account. In this investigation, most of the quantitative data were retrieved from chemical risk assessment reports, where a peer-review evaluation of toxicological studies was already conducted, and therefore the related data quality was assumed to be high. Thus, for the quantification of the AOP, the relevant dose–response data for quantitative characterisation of the response-response function between different KEs and the AO were collated. Since all datasets were from animal studies (due to the selection of KERs for quantification), the study design and associated dataset were carefully reviewed.

The relevance of having well-defined inclusion criteria for studies to be used for quantification was acknowledged. Another key learning was the type of dose–response information to be used: preferably, quantification of KERs should be based on datasets that include KEs at multiple levels of biological organisation and with significant increases over controls for at least two doses/concentrations. Finally, species/sex and strain, differences in exposure period and exposure routes was recommended. The case study made very clear the lack of uniformity in data reporting among the different data sources, contributed to significant gaps and consequent uncertainty.

#### Modelling approaches

For modelling of the carcinogenicity pathway, an empirical dose–response model as described in Zgheib et al. [31] was applied. In addition, a physiologically based kinetic (PBK) model was used to account for different routes of exposure between different animal studies [32]: application of the PBK model for calculating the internal dose allowed for comparison of the different datasets.

## Workflow to support qAOP development

**Fig. 3:**
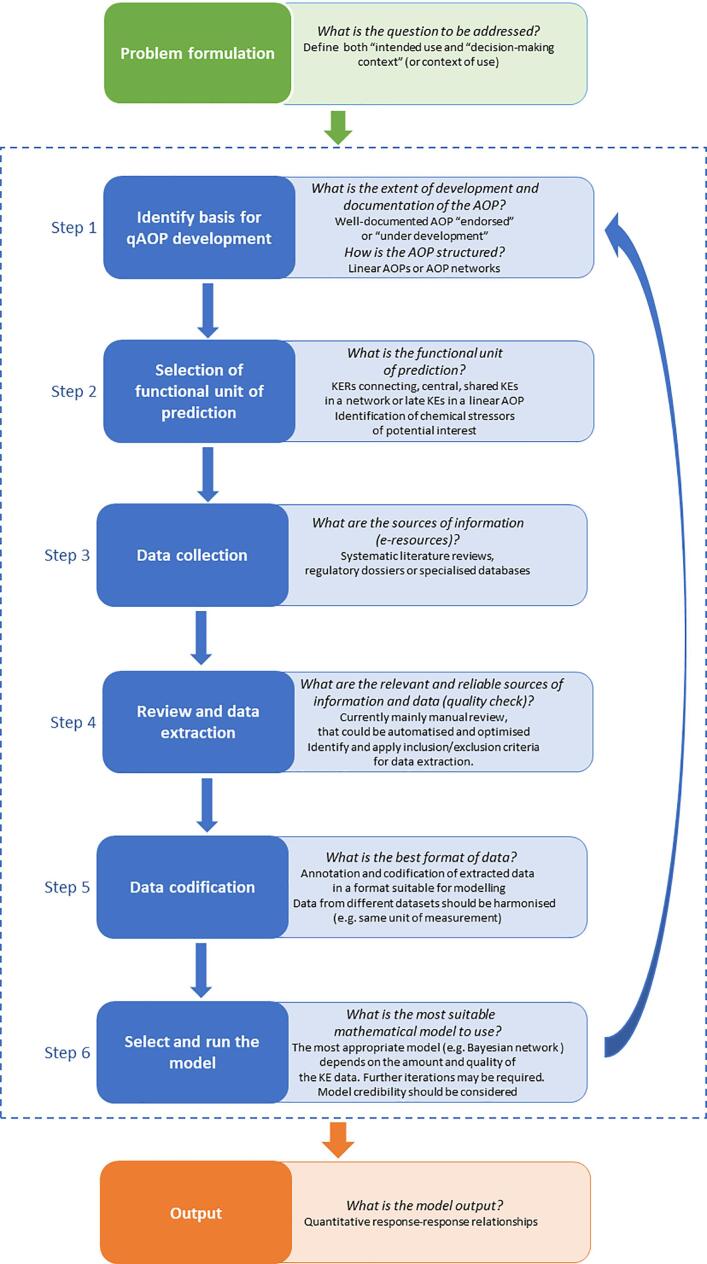
General stepwise approach proposed for qAOP development.

Based on the key findings and learnings from the three case studies (Section 2, Table 1), a generic workflow consisting of six steps is proposed (Fig. 3) to support qAOP development. The workflow is intended to be applied in an iterative manner until sufficient confidence in the development of the qAOP is achieved.

Before entering the workflow to design a qAOP, the scope and purpose (problem formulation) should be defined. The problem formulation is an iterative process involving risk assessors and risk managers who determine the need for, and the extent of, a risk assessment [40]. It is important to ensure that the question(s) are clear, specific and agreed upfront before developing the qAOP. Relevant considerations include the decision context, critical issue(s) that need to be addressed, and the required level of confidence in outcome, including any quantitative modelling [41]. In this context, “intended use” refers to the scientific purpose of the model (e.g., generation of a dose-metric and its use in a risk assessment), while the “decision-making context” (or context of use) refers to relevant considerations (e.g. acceptable uncertainty, risk management consequences of making a decision, availability of existing data, possibilities to generate new data, restrictions/bans on animal testing) [42]. Once the problem formulation is defined, the workflow can be executed to address specified objectives.

The **first step**, of the workflow (Fig. 3) consists of identifying the basis for qAOP development, i.e. a single AOP (in the case of skin sensitisation, and carcinogenicity for example) or an AOP network (in the case of neurotoxicity for example) [2], [43]. According to what is considered the required functional unit of prediction (**step two)**, the modelling challenge is simplified by selecting the “more important or more relevant” nodes in the AOP or AOP network. The choice of nodes (KEs) in a simplified network can be informed by network analytics. The degree of connectedness and centrality of KEs can express the extent to which the KEs represent important nodes of convergence of the pathway, in the context of the problem formulation [17]. Information on highly-connected KEs from network analytics needs to be weighed against the availability and quality of data, as well as the cost-effectiveness of generating new experimental data. **Step three** consists of identifying sources of information (from the literature and/or databases) that contain potentially useful data for quantifying the KERs that will be derived from pairs of KEs. The datasets should meet the FAIR data principles [19] and interventions at both data and metadata level could support this FAIRification process for newly created or existing datasets (https://www.go-fair.org/fair-principles/fairification-process/).

The **fourth step** consists of reviewing the information and extracting that considered to be reliable and relevant. At present, this is a manual and time-consuming process, but this could eventually be partially automated. For example, steps 3 and 4 could be facilitated by using online e-resources such as Abstract Sifter [44], Swift Review [45], DistillerSR [46] (see [47] for additional examples). Easy access to the protocols underlying the data and the definition of the inclusion and exclusion criteria for studies and associated datasets would also facilitate the review and extraction of the data. In the OECD programme, the value and potential modification/application of tools for the systematic consideration of available data are being considered as a basis to increase efficiency and transparency in AOP development, paving the path for new Artificial Intelligence tools to be developed to facilitate data extraction. Recent activities are oriented to promote the use ontology-based annotations of KEs and establish semantic mapping of AOP Wiki, to facilitate the extraction of AOP content, support computational analysis and allow better interoperability with other resources [48], [49], [50], [51] (see Supplementary Material).

In the **fifth step,** the (manually) extracted data are codified and annotated in a machine-readable format, suitable for modelling. Attention should be paid to relevant meta data, including the correct units of measurement.

The **sixth step** consists of building and running the mathematical model (i.e. the qAOP). The choice of modelling approach will depend on the amount, quality and comparability of the data on incidence, prevalence of the KEs, whereas the choice of software platform will depend on the preferences of the modeller. Moreover, the selected model may depend on the required degree of parametrisation, considering that data may not always be sufficient or data processing could be resource-intensive. Model credibility should be considered. Several guidance documents illustrate how to make mathematical models more credible [40], [41], [42], [52].

Finally, the model outputs are reported. Depending on the uncertainties in the model simulation, the model results will either be used directly to inform the hazard assessment, or there will be a further iteration to generate data or refine the model to reduce the uncertainties and build more confidence in the qAOP. It is important that the model outputs are useful for decision making (in the sense that they provide information hat meaningfully informs the decision context.

The overall process should be reported in a transparent way, indicating the assumptions and limitations for each step. The application of a framework for qAOPs would provide the basis for deciding when the model is adequate for the intended purpose (Section 5).

## Qualitative and quantitative understanding of AOPs

This section presents elements of AOP description and assessment that are relevant to the workflow proposed for qAOP development. Qualitative weight of evidence considerations and quantitative understanding of the KERs in an AOP facilitate early focus on the generation of data, considerations of e-resources and their possible applications to quantification in early stages of development and assessment of AOPs.

### Weight of evidence considerations

The Weight of Evidence (WoE) analysis in AOP development is a transparent judgment concerning the extent of the supporting evidence. This analysis is based on guidance outlined in the OECD guidance and Handbook for AOP development, drawing upon well-established considerations in the assessment of mechanistic data on chemicals by regulatory agencies [5], [7], [9], [28] (https://aopwiki.org/). The WoE analysis communicates the extent of supporting knowledge for AOPs in a format which supports quantitative and regulatory application and promotes better common understanding within and between the research and regulatory communities on the types of data or studies that are most informative. WoE analysis is based on a subset of modified Bradford Hill (B/H) considerations, which were introduced in 1965 to assess the causality of associations in epidemiological studies [53]. These considerations have evolved based on international experience in applying Mode of Action (MOA) analysis for regulatory applications [54], [55].

The modified or tailored B/H considerations for AOP analysis are “expert informed” and application driven, reflecting additional evolution taking into account experience in the OECD AOP development programme. They focus on those aspects that are considered most critical to ensure adequate documentation for regulatory application, while recognising the needs of developers for simplicity in communicating their critical knowledge of biology. The relevant subset of considerations are, in rank order of importance, biological plausibility, essentiality and empirical support [5], [6], [7]. Delineation of relative confidence in the supporting evidence for each of these considerations for KEs, KERs and the overall AOP informs the extent of the robustness of AOPs for application (for example, in the development of testing strategies and/or as a component of priority setting or hazard characterisation).

**Fig. 4:**
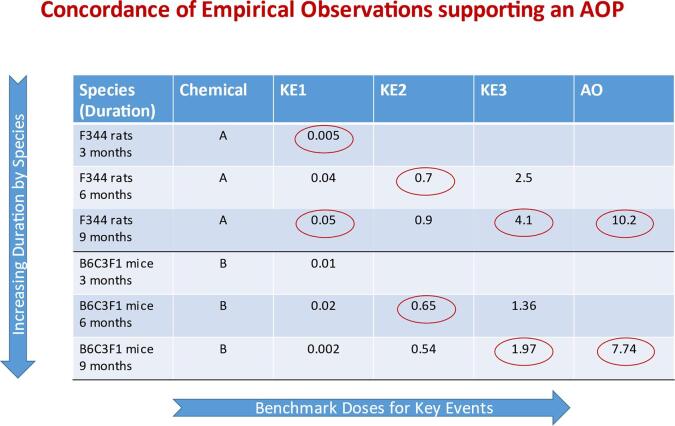
Dose-Response and Temporal Concordance Table illustrating empirical support for a hypothesised AOP. Data are considered independently for different stressors (Chemicals A and B) and different species (rats and mice) and presented by increasing duration of exposure (for both rats and mice). Chemicals A and B are thought to act on the same MIE. Benchmark doses for key events are presented to see if they align, based on the expected pattern. In this example, the empirical data fully support expected relationships across KERs for the hypothesised AOP (i.e. increasing values from the upper left corner to lower right hand corner, illustrated by the redlined circles).

**Fig. 5:**
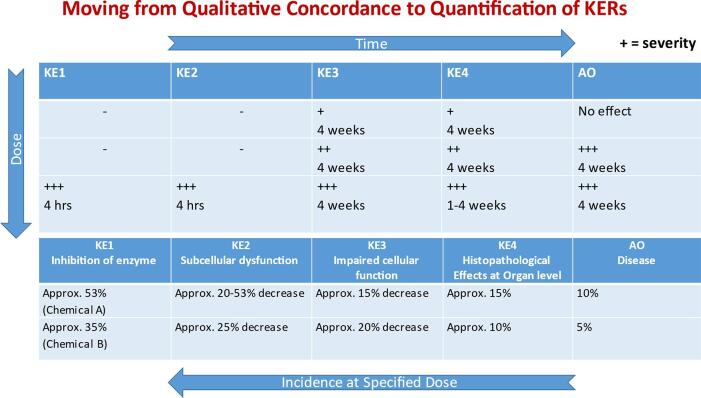
Relationship between empirical support for a hypothesised AOP and quantification of the KERs. The dose–response and temporal concordance table at the top addresses severity over time, in contrast with Fig. 4, which addresses benchmark doses. Dose-response and temporal concordance tables address various measures of dose–response, depending upon the nature of the data reported. The number of plus signs indicates the degree of severity of the observed effect - + = low, + + = moderate, + + + = high. The lower table addresses the incidence of the effect at a specified dose, and provides information relevant for the quantification of KERs. Chemicals A and B are thought to act on the same MIE.

Concordance tables that summarise critical information pertinent to the expected patterns between the upstream and downstream event(s) can be very effective in facilitating WoE evaluations (Fig. 4). They are also critical in identifying the information relevant to subsequent quantification of the KERs (Fig. 5). These WoE determinations contribute to the consistency and transparency of AOP descriptions. They are also helpful in characterising uncertainty, including the nature of gaps in biological knowledge of relationships between KEs and limitations in the extent of investigation of empirical support for KE essentiality and for KERs.

As a result, the WoE considerations increase common understanding among the research and regulatory communities of the elements and types of data or studies which increase confidence for regulatory application of AOPs. They constitute the essential bridge between development and application of AOPs. While these considerations are qualitative in nature, empirical support (i.e., expected patterns of quantitative relationships across the KERs) focuses early attention on dose–response information for KEs that have potential to contribute to the quantification of KERs, as a basis for developing quantitative response-response models (i.e., to characterise how much change in an upstream KE is needed to evoke some unit of change in a downstream KE). For cases where there is a preponderance of high and/or moderate confidence determinations for empirical support, it is likely that data are sufficient to support development of quantitative models.

Extension of qualitative characterisation of relative WoE for AOPs based on specified considerations has also been proposed, with the aim of establishing a quantitative approach for WoE evaluation of AOPs (for example, [56], [6]). This would allow for WoE analysis of complex datasets with multiple criteria and metrics and would be particularly useful for more complex AOPs or AOP networks. Such analysis is potentially more relevant in chemical hazard characterisation where there may be a need to consider more quantitatively the extent of supporting evidence for different hypothesised modes of action in the induction of adverse outcomes. Quantitative WoE approaches are based directly on the rank ordering of the qualitative WoE considerations through assignment of relative scores. While the assignment of specific scores to the various considerations is necessarily arbitrary, it should be sufficient to characterise the extent of supporting data in a relative rather than absolute context. As a result, quantitative scores for AOPs or their components can only be interpreted in the context of their magnitude relative to scores for other components or AOPs, respectively. It should be noted that careful consideration should be given to the extent to which the components of the AOP have been studied. Lower relative scores do not necessarily indicate a lower probability of an event occurring, but may reflect a simple data gap.

Qualitative WoE provides, then, a measure of confidence in AOP elements and the overall AOP, based on the extent of the supporting data. The considerations are rank ordered with biological plausibility being most important, followed by the essentiality of key events. Empirical support is the least influential of the three considerations related to correlation contributing less to consideration of causation. Rather, empirical support contributes, along with biological plausibility and essentiality. A small amount of empirical support can provide high confidence, if there is high confidence in biological plausibility of the KERs and essentiality of the KEs. However, if there is weak support for plausibility and essentiality, much more empirical support is required for predictive confidence. While qualitative consideration of empirical support is distinguished from quantitative modelling of response-response relationships that form the basis for predictive dose–response models, they are based on the same or similar data. In particular, specific studies identified in the concordance tables or text summaries for empirical support for each of the KERs and AOP overall should be helpful in the quantification of response-response relationships. Indeed, an extension of the concordance tables to include additional detail relevant to quantification of the dose–response relationships is likely to be helpful in informing quantitative modelling of the KERs (Fig. 5).

Within qualitative consideration of WoE for hypothesised AOPs, empirical support takes into account “patterns” of quantitative relationships for KERs (i.e., the extent to which temporal and dose–response patterns align with what would be anticipated for key events). These patterns are normally considered on the basis of analysis of dose–response relationships for KEs across different levels of biological organisation following exposure to stressors which perturb the pathway. This differs from quantification of the KERs, i.e., the quantification of the extent of change in an upstream KE (KEup) needed to evoke some unit of change in a downstream KE (KEdown). It is quantification of these KERs which lends itself to development of predictive response-response models.

The extent of development and required accuracy of such models is necessarily dependent on the purpose-specific application, as framed in the problem formulation. Although not yet formally addressed, it can be anticipated that the evolution of predictive quantitative modelling of AOPs will include the development of guidance for the description and application of purpose-specific quantitative models of KERs and AOPs, consistent with principles outlined in previous initiatives, for example, on PBK models [40], [42], [57]. Key considerations in the description of such models, beyond the qualitative consideration of the extent of supporting evidence for AOPs addressed above, are discussed in Section 5.

### Quantitative understanding of key event relationships

There are four types of information that AOP developers are encouraged to document to support quantification of KERs; each of these is discussed in more detail below. The information required is often included in studies considered in assessment of empirical support for the KERs.

The first (i), and arguably most important of these, is **a** quantitative response-response relationship. A quantitative response-response relationship can be viewed as a mathematical function or model that allows one to address the question – how much change in KE (A) is needed to evoke some defined level or magnitude of response in downstream KE (B) [58]? A quantitative response-response relationship is analogous to the concept of dose–response familiar to most toxicologists. However, rather than identifying dose (concentration) of a stressor as the independent variable, the magnitude of change in key event A is the independent variable, and the magnitude of change in downstream key event B is the dependent variable. In the case of quantitative response-response relationships, there is no expectation that this function will follow the typical sigmoidal shape of a dose–response curve. Rather, it may be described by a wide range of functions that may range from simple linear or non-linear regressions, to rather complex biologically-based models employing systems of ordinary differential equations that may capture non-intuitive behaviour and even stochasticity (e.g., [14], [15], [59], [60]).

The second (ii) aspect of quantitative understanding of a KER is an understanding of the **time-scale** over which key event A can be expected to impact key event B. Generally speaking, the time-scales of biological events tend to increase as one moves to higher levels of biological organisation, with most events on the molecular and cellular level happening on scales of seconds to minutes, events at the tissue and organ level happening on scales of days or weeks, and effects on individuals or populations manifesting on scales of months or even years. Understanding the time-scale of a transition from one key event to the next is important for many applications. For example, it may inform what measurements are practical to make experimentally, versus those that may be best addressed through modelling (i.e., happen very rapidly or on the scale of years). It can provide critical insights when evaluating evidence that may support or reject the empirical concordance between two key events, as described above. Furthermore, it can inform both the type of evidence one might expect to find in relation to the KER and how one would design an experiment to evaluate the response-response relationship defined for a given KER. For example, if the transition from one KE to the next in a KER occurs on the scale of years, epidemiological lines of evidence may be far more tractable than experimental lines of evidence. Consequently, the time-scale of the transition is viewed as another important component of the quantitative understanding of any KER.

The third (iii) aspect of quantitative understanding of a KER is identification of known modulating factors. A critical question involved in applying the quantitative response-response relationship identified for a KER is “how generalisable is this relationship?” Is the relationship valid for just a single species under very specific test conditions, or can it be extended to other organisms? Does it apply to all life stages, or only to a specific life stage? In part, these questions are addressed in the domain of applicability of a KER, which seeks to define the species, sexes, and life stages for which a given relationship between key event A and key event B is expected to hold up. However, in a quantitative sense, other factors come into play: it also entails defining how different factors intrinsic to the organism and its biology, as well as extrinsic factors acting on the organism (collectively termed modulating factors), may alter the response-response relationship defined for the KER. Modulating factors may include genetic background, diet, pre-existing disease states, environmental stresses, social stresses, and many others.

Some modulating factors may make event B much more sensitive to changes in event A. Other modulating factors may make event B less sensitive or even completely insensitive to event A. Still others may dramatically alter the shape of the function describing event A’s effect on event B. Whatever the case, where this information is known, AOP developers are encouraged to summarise and support information on these modulating factors and their influence on the response-response relationship for the KER, where possible. However, AOP developers are discouraged from merely speculating as to the role of various modulating factors. Identification and characterisation should be founded on empirical scientific support, not just plausibility.

The fourth (iv) and final consideration in documenting the quantitative understanding of a KER is the consideration of any feedback relationships, positive or negative, that may exist between events A and B. In the typical box and arrow diagram used to depict an AOP, the upstream event (event A) is assumed to cause a change in the downstream event (event B). The conceptual representation implies that the state of event A is independent of any change event B. However, biologically it is understood that event A may indeed be impacted by changes in event B. For example, in a series of studies examining the relationship between aromatase inhibition (a MIE) and decreases in circulating concentrations of 17β-estradiol (E2), it was consistently shown that decreases in circulating concentrations of E2 are followed by increased expression of mRNA transcripts coding for aromatase, and subsequent partial recovery in E2 concentrations as exposure persists [61], [62], [63], [64]. Behaviours associated with this feedback relationship between events A and B were incorporated into the models used to derive a response-response relationship linking aromatase inhibition to reduced circulating E2 [14]. The publication by Knapen et al. [65] provides another example in which a downstream event not only influences the mRNA expression related to an upstream event, but the feedback mechanism itself involves a biological object represented in other AOPs. Thus, capturing feedback can be critical not only to quantitative application of individual AOPs, but also the use of AOP networks in predictive toxicology [65]. Where this kind of quantitative understanding can be assembled for KERs that span across the KEs and levels of biological organisation represented in an AOP, the AOP may support a much broader range of applications and decision-making [2], [59], [60].

## Four groups of e-resources

**Fig. 6:**
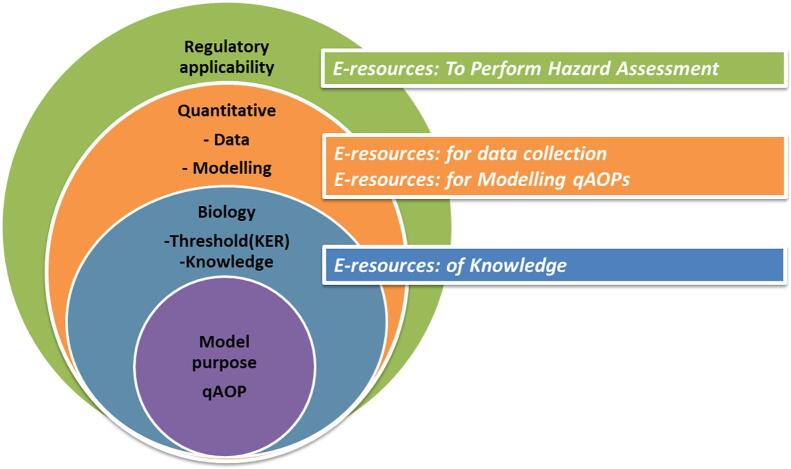
Graphical summary representing the elements described in the present paper, from qAOP model purpose, through knowledge of biology and quantification of the KE by measured data to modelling predictions driven by regulatory application. For each step e-resources are mapped.

A wide variety of electronic resources that may contribute to the development of qAOPs are available. Spinu et al. [16] published an extensive review of tools and resources to support qAOP development. These resources can be categorised into four primary types: knowledge, data, modelling, and resources to perform hazard and safety assessment (Fig. 6). For each step, relevant e-resources have been mapped. A comprehensive but not exhaustive list of e-resources provided by the experts during the workshop are available in the Supplementary material (Supplementary Table 1).

### Knowledge

Quantitative AOPs are knowledge-based algorithms and models, with the supporting descriptions being documented in frameworks of mechanistic toxicology. The workshop considered a number of knowledge sources to support the development of qAOPs, from the AOP-Wiki providing AOP descriptions to the underlying literature and data sources. Abstract Sifter [44] is one such example, along with a variety of other text mining tools (see Supplementary Table 1), that could aid extraction of biological and toxicological knowledge.

### Data

Data are required to populate qAOPs, ranging from *in vitro* data through to *in vivo* toxicity and observations of adverse effects. There are numerous resources and data compilations. For example, Pawar et al. [18] reported nearly 1,000 toxicological data resources that may assist in modelling. Fundamental issues that must be considered when using literature data are the quality of the information in terms of the veracity of chemical structure, intrinsic quality and relevance of the original study and that data have been captured correctly, i.e. no errors in transfer. Thus, the quality and accuracy of the data must be checked by the user. There is need for adoption of FAIR data sharing principles, along with the provision of (meta)data that accurately describe biological and toxicological relevant endpoints of the studies. Some of the key data resources can complement the qualitative description of AOPs available through the AOP-Wiki (https://aopwiki.org/) and may be utilised to populate qAOPs, for example, JRC EURL ECVAM datasets [66], the OECD QSAR Toolbox [33], OpenFoodTox [36], eChemPortal [35], US EPA AOP-DB [49] and [81], US EPA CompTox Chemicals Dashboard [67] or PubChem [68] (see Supplementary Table 1 for additional information).

### Modelling

Implementation of a computational qAOP model generally requires some form of modelling software to assist with the mathematical description and analysis (see Supplementary Table 1 for details on software and availability). A variety of resources for quantitative modelling of KERs is available. These include freely available resources and those that require commercial licenses.

Software environments such as R (https://www.r-project.org/) and Python (https://www.python.org/) are readily usable and provide packages which are transferable for the purpose of developing qAOPs.

The workshop also considered several other bespoke resources which have been developed for modelling in systems biology, biokinetics and for decision analysis such as DART (Decision Analysis by Ranking Techniques) [69]. Freely available software (such as KNIME [70]) can incorporate these statistical models into usable computational workflows. There are also resources that can assist with the evaluation of literature and more efficient mining of text information. Other resources were also considered such as reported by Madden et al. [71] who provided a detailed overview of computational resources for the parameterisation of PBK models. PBK models are mathematical description of the adsorption, distribution, metabolism and excretion of chemicals in the body and are usually described by ordinary differential equations. This is complementary to a qAOP model which is a mathematical description of the mechanistic downstream events/mode of action of the chemical after the kinetic process has taken place.

### Hazard and safety assessment

The translation of qAOPs to usable tools to assist in hazard and safety assessment is essential for their uptake in the broader scientific community. Currently there is no platform that provides a systematic integration of qAOPs for use in hazard and safety assessment. However, the workshop noted some existing resources, such as the OECD QSAR Toolbox [33], EFSA Knowledge Junction [72] and Euromix Toolbox [73], as useful now and in the future for this purpose.

Several different software tools (Supplementary Table 1) are available and although the choice mostly depends on the modeller’s preferences, it is advisable to use open-source applications that increase transparency, transferability and acceptance of the model. Confidence in qAOP models may increase through iterative refinement of the model for example by developing more comprehensive datasets, which many imply the need to conduct additional studies. It is also possible that the inclusion of additional data will result in the need to refine the original model (choice of KEs and KERs). In addition, challenging the model with reference compounds that were not used for the initial model calibration increases confidence in the results. An electronic infrastructure such as the Adverse Outcome Pathway Knowledge Base (AOP-KB, https://aopkb.oecd.org/background.html) provides a perfect host platform to implement automated qAOPs in a user-friendly, open access environment.

The suggested modelling strategy is to consider the question of interest and then search the AOP-Wiki for KERs of relevance, considering that quantitative data are more likely to exist or to a larger extent for KERs for which empirical support in qualitative weight of evidence calls is “High”, and also that KERs at higher levels of biological organisation (i.e., closest to the adverse outcome) are likely to be of greatest interest currently for the regulatory community for hazard characterisation. In fact, later key events are less chemical specific and more often an expected consequence of progression of earlier key events (e.g., regenerative proliferation resulting from cytotoxicity). Focus on human health risk assessment has traditionally been on (typically late) key events that provide quantitative information relevant to intraspecies and interspecies extrapolation and life stage susceptibility for dose–response analysis [55]. For development of integrated approaches to testing and assessment, earlier key events are also important.

In addition, effort will need to be placed into better understanding of the level of uncertainties that may be appropriate and or acceptable to make a regulatory decision based on the qAOP, thus interpretation of the information is essential. There are several sources of uncertainties, from the input experimental data, to the modelling parameters and equations. These sources of errors should be listed and evaluated when considering the final results. However, questions regarding model uncertainty in qAOP are still underexplored, highlighting the need to develop a rational framework for evaluating this uncertainty. At the present moment, these uncertainties should be documented and reported by following available guidance [40], [41], [42].

## Towards an assessment framework for qAOPs

An important discussion point during the workshop concerned the challenges involved not only in developing qAOPs, but also in promoting their acceptance and use, especially for regulatory decision making. While a qualitative AOP may be sufficient for hazard identification, an adequate qAOP (or at least modelling of certain KERs) will be needed for hazard characterisation and risk assessment [15]. The question however is what constitutes an “acceptable” or “credible” qAOP. Recognising that model credibility is the “*willingness of others to use predictions to inform decisions*” [74], it was argued that model credibility is ultimately subjective, depending on the end-user and a range of contextual factors. In addition to the inherent scientific validity of a model, contextual factors include context of use, as well as social, economic and political factors. The context of use would include the regulatory purpose and constraints (e.g. ban on animal testing) under which decision making is made, but also whether the qAOP model is used on its own, or in the context of a tiered assessment strategy, such as that proposed by Berggren et al. [75].

**Fig. 7:**
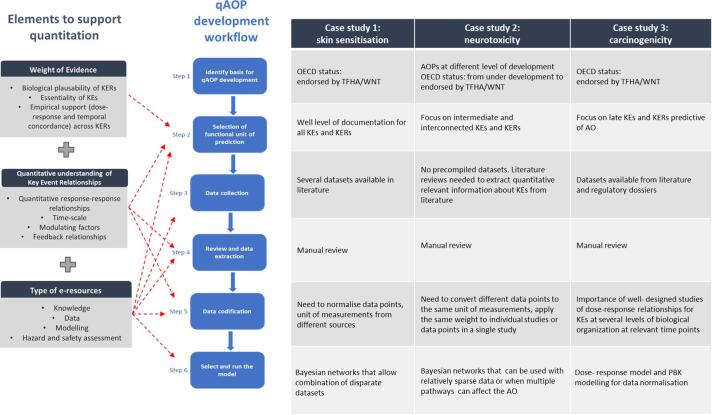
Characteristics of a Framework for qAOP development. The major elements - weight of evidence, quantitative understanding of KERs and e-resources - that support AOP quantification are indicated. Red dotted lines show examples of how these elements can contribute to different steps of the qAOP development workflow. The table summarises the steps of the qAOP development workflow for the three case studies. (For interpretation of the references to colour in this figure legend, the reader is referred to the web version of this article.)

Hence, there is a need to guide qAOP developers, scientists and regulators by laying out a modelling workflow and assessment framework for qAOPs. During the workshop, it was argued that a harmonised assessment framework will be needed to support the regulatory applications, acknowledging however that not all of the contextual factors can be included in the framework. Fig. 7 shows several elements of a possible assessment framework for qAOP development, challenged by the three AOPs with a different basis (linear/network) for different endpoints at different stages of OECD endorsement. Three types of elements – Weight of evidence, quantitative understanding of KERs and e-resources - support quantification of AOPs at different steps of the proposed workflow (Section 4).

Logically, the credibility of a qAOP model will depend on the confidence in the underlying AOP (or AOP network), as well as the confidence in the mathematical modelling approach. In turn, confidence in an AOP can be deconstructed into confidence in the data quality for the KEs as well as confidence in the causality of the KERs (Section 4.1). A starting point for the data quality of key events could be the potential application of the OECD GIVIMP [22] and reporting of mechanistic data using the OECD harmonised template (OHT) on intermediated effects (OHT 201, [76]). While confidence in the KERs could be established by using the evolved Bradford Hill considerations [7], [54]. A standard reporting template or guidance for reporting should be created and shared among regulatory bodies and companies to ensure harmonised reporting of the qAOP model and underlying data. As a basic indication, individual empirical data and associated statistics should be clearly reported. Empirical data should be generated for mechanistic key points, to supplement data for traditional (apical) endpoints.

A challenge in establishing an assessment framework for qAOPs is the diversity of modelling approaches, which may include for example Bayesian models [77] or systems biology models [78]. It was further proposed that model-dependent elements of the assessment framework could be inspired by existing frameworks. In particular, probabilistic models could be assessed in essentially the same way as QSAR models (e.g [79]), while mechanistic models could be treated like PBK models [40], [42], [57], [80]. Generally, models intended for use in regulatory decision making should be scientifically sound, robust, thoroughly tested and make valid predictions [41]. Meeting these expectations requires a level of accuracy that can only be guaranteed by using experimental data of sufficient quality and quantity to support the level of certainty required [78]. Key considerations in the description of such models, beyond the qualitative consideration of the extent of supporting evidence for AOPs addressed above, may include sufficient information to enable reproduction of the input–output relationships, a mathematical description of the qAOP model and the methodology underlying its development, evidence of computational implementation and aspects of model verification and validation.

## Concluding remarks

In the past decade there have been several innovations to improve chemical risk assessment, including new terms that have been coined to facilitate communication between scientists and the regulatory risk assessment community. One of these innovations is the AOP concept. It evolved from mode of action experience to provide a more predictive context leading to the development of a knowledge resource (AOP-KB) for information on biological pathways to disease including underpinning *in vivo*, *in vitro*, and *in silico* data. The next step is to provide an automated means of applying this knowledge to better inform hazard characterisation and risk assessment by mathematically modelling the KERs of an AOP. Quantification of an AOP can have two aspects, including not only the measurements for adjacent KEs (i.e., KERs) by biological methods (e.g. concordance of concentration response curves), but also the mathematical prediction of the function. Both aspects are needed to evolve the e-resource from a source of knowledge to an e-tool which will enable the execution of the full AOP, or parts of it, as an automated process. Understanding the assumptions and capabilities of different kinds of qAOP models is essential not only for those interested in modelling an AOP, but also for those interested in using the resulting qAOP models. The proposed framework will facilitate this uptake and the workflow should be applied in an iterative manner until sufficient confidence in the development of the qAOP is achieved.

In addition to providing the necessary e-resources, tools and guidance for qAOP development, a further challenge in transitioning to more predictive and efficient approaches in chemical risk assessment is to train the next generation of researchers, risk assessors and regulators to move away from the current animal testing based paradigm to one that is centred around knowledge, modelling and digital resources (e-resources) capturing biological responses at different levels of biological organisation.

## Declaration of Competing Interest

The authors declare that they have no known competing financial interests or personal relationships that could have appeared to influence the work reported in this paper.

## Footnotes

3 Task Force on Hazard Assessment / Working Group of the National Coordinators for the Test Guidelines Programme
