# Supplementary material for: Towards a qAOP framework for predictive toxicology - Linking data to decisions
Source: Comput Toxicol. 2022 Feb;21:100195. doi: 10.1016/j.comtox.2021.100195 (PMC8850654; doi:10.1016/j.comtox.2021.100195)
Supplement: Supplementary data 1 [file mmc1.docx]

**Title:** **‘Towards a qAOP Framework for Predictive Toxicology - Linking Data to Decisions’**

**Authors:** Alicia Paini^1, ¥^, Ivana Campia^1,¥^, Mark T.D. Cronin^2^, David Asturiol^1^, Lidia Ceriani^3^, Thomas E. Exner^4, #^, Wang Gao^5^, Caroline Gomes^6^, Johannes Kruisselbrink^7^, Marvin Martens^8^, M.E. Bette Meek^9^, David Pamies^10^, Julia Pletz^2^, Stefan Scholz^11^, Andreas Schüttler^11^, Nicoleta Spînu^2^, Daniel L. Villeneuve^12^, Clemens Wittwehr^1^, Andrew Worth^1*^, Mirjam Luijten^13^

**Affiliations:**

^1^European Commission, Joint Research Centre (JRC), Ispra, Italy

^2^Liverpool John Moores University, Liverpool, United Kingdom

^3^Humane Society International, Brussels, Belgium

^4^Edelweiss Connect GmbH, Technology Park Basel, Basel, Switzerland

^5^Institut National de l'Environnement Industriel et des Risques (INERIS), Verneuil-en-Halatte, France

^6^BASF, Ludwigshafen, Germany

^7^Wageningen University & Research, Wageningen, The Netherlands

^8^Maastricht University, Maastricht, The Netherlands

^9^University of Ottawa, Ottawa, Canada

^10^Department of Physiology, Lausanne and Swiss Centre for Applied Human Toxicology (SCAHT), University of Lausanne, Lausanne, Switzerland

^11^Helmholtz Centre for Environmental Research GmbH – UFZ, Leipzig, Germany

^12^US Environmental Protection Agency, Great Lakes Toxicology and Ecology Division, Duluth, Minnesota, USA.

^13^ Centre for Health Protection, National Institute for Public Health and the Environment (RIVM), Bilthoven, The Netherlands

^#^Current affiliation: Seven Past Nine d.o.o., Cerknica, Slovenia

*Corresponding Author: andrew.worth@ec.europa.eu

^¥^ Equal contribution

**Supplementary material**

Supplementary Table 1: list of e-resources available identified by the experts during the Lorentz workshop in October 2019.

Classification legend - 4 types of e-resources-Data =D; Modelling=M; Knowledge = K; Assessment Tool = AT.

| **e-resource**  **name** | **Description** | **Potential application in qAOP** | **e-tool reference/link** | **Type of e-resources** |
| --- | --- | --- | --- | --- |
| AOP-DB- Adverse Outcome Pathway Database | The AOP-DB is currently an internal EPA SQL database that supports discovery and development of putative and potential AOPs. The AOP-DB aggregates relationships between AOPs, gene targets, chemical, disease, tissue, pathway, species orthology information, ontologies and gene interactions to characterise the impacts of chemicals on human health and the environment. | Gaining information on chemicals and biology (input data) | [1] | D |
| AOP-DB SPARQL Endpoint | Virtuoso SPARQL endpoint that is loaded with RDF of the EPA Adverse Outcome Pathway Database (AOP-DB) from the US EPA. | Tool to query the AOP-Wiki content through web services, coding environments and computational workflows, and linking with other databases. | ttps://openrisknet.org/e-infrastructure/services/147/ | K-D |
| AOPWiki Resource Description (RDF) Framework and SPARQL endpoint | The AOP-Wiki Resource Description Framework is a semantic model of the AOP-Wiki to facilitate accessibility and interoperability of AOP-Wiki content, with added molecular descriptors and ontological annotations. | Tool to query the AOP-Wiki content through web services, coding environments and computational workflows, and linking with other databases. | <https://aopwiki.rdf.bigcat-bioinformatics.org/>  [2] | K-D |
| Chemchart | Chemchart is an AI-powered platform for chemical industry solutions. We combine big data and machine learning to connect industry with actionable chemical information. | Gaining information on chemicals (input data) | <http://api.chemchart.com/> | D |
| DART - Decision Analysis by Ranking Techniques | Freely available software implementing partial order ranking and several total ranking methods | A tool for ranking objects (e.g. chemicals) for priority setting or data gap filling (interpolation) | <https://ec.europa.eu/jrc/en/scientific-tool/dart-decision-analysis-ranking-techniques> | M |
| eChemPortal | eChemPortal provides free public access to information on properties of chemicals: –Physical Chemical Properties –Ecotoxicity –Environmental Fate and Behaviour –Toxicity | Gaining information on chemicals (input data) | <https://www.echemportal.org/echemportal/index.action> | D |

| **e-resource**  **name** | **Description** | **Potential application in qAOP** | **e-tool reference/link** | **Type of**  **e-resources** |
| --- | --- | --- | --- | --- |
| ECOTOX | The ECOTOXicology knowledgebase (ECOTOX) is a comprehensive, publicly available knowledgebase providing single chemical environmental toxicity data on aquatic life, terrestrial plants and wildlife. | Information data on chemical environmental toxicity data on aquatic life, terrestrial plants and wildlife. (input data) | <https://cfpub.epa.gov/ecotox/> | D |
| Effectopedia | Effectopedia is an open-knowledge and structured platform able to display quantitative information on Adverse Outcome Pathways (AOPs). Effectopedia is a tool for visual exploration and development of AOPs compliant with OECD Users’ Handbook. | Part of AOP wiki: The AOP diagram captures the causal linkages between AOP elements in their biological context. (model repository) | <https://www.effectopedia.org/> | K-D |
| EURL ECVAM Datasets - JRC catalogue | Databases and information systems on alternative approaches | Data available for in vitro intrinsic clearance, biotransformation, genotoxicity. Information on nanomaterials. | <https://data.jrc.ec.europa.eu/collection/id-0088> | D |
| Euromix Toolbox | Web-based toolbox for mixture risk assessment | Monte Carlo Risk Assessment (MCRA) toolbox, also known as the EuroMix toolbox, modular model and data toolbox to assess combined chemical exposure risks | <https://mcra.rivm.nl>  [3] | AT |
| Github | Tools for online qAOP storage and sharing | Model repository | <https://github.com/> | K |
| High-Throughput Toxicokinetics (httk) | R package for simulation and statistical analysis of chemical toxicokinetics | Modelling of toxicokinetics | <https://cran.r-project.org/web/packages/httk/index.html> | M |
| KNIME | software to create WF to use models and data science in one easy and intuitive environment | qAOP could be built within KNIME such as one node may represent one KER. Furthermore, nodes can embed scripts of programing language such as R, Python, Matlab etc. These scripts could host complex models such as Bayesian networks embedded. | <https://www.knime.com/> | M |

| **e-resource**  **name** | **Description** | **Potential application in qAOP** | **e-tool reference/link** | **Type of**  **e-resources** |
| --- | --- | --- | --- | --- |
| Matlab/SimBiology (desktop - not an online tool) | programing languages | Can be applied to develop models and qAOP. By targeting each individual KE - KER but also linking them together. (Model code) | https://www.mathworks.com/products/matlab.html - https://www.mathworks.com/products/simbiology.html | M |
| MCRA 9 –EuroMixtoolbox | web-based system for human health hazard identification, hazard characterisation, exposure assessment and risk assessment related to chemical substances. MCRA stands for Monte Carlo Risk Assessment. The MCRA system brings together statistical models, shared data and data uploaded by the user. | Can help in co-exposure, IVIVE, and incorporate Monte Carlo analysis (downstream analysis such as MC) | <https://mcra-test.rivm.nl/EuroMix/WebApp/#/> | M |
| OECD QSAR Toolbox | The Toolbox is a software application intended to the use of governments, chemical industry and other stakeholders in filling gaps in (eco)toxicity data needed for assessing the hazards of chemicals. The Toolbox incorporates information and tools from various sources into a logical workflow. Crucial to this workflow is grouping chemicals into chemical categories. Download our brochure (PDF). | Helps into incorporate information for qAOP development, such as filling data gaps (Data gap filling, input data) | <https://www.oecd.org/chemicalsafety/risk-assessment/oecd-qsar-toolbox.htm> | D-AT |
| OpenFoodToxdatabase | Chemical hazard data - OpenFoodTox provides open source data for the substance characterisation, the links to EFSA’s related output, background European legislation, and a summary of the critical toxicological endpoints and reference values. | Information on chemical hazard data (input data) | <https://www.efsa.europa.eu/en/microstrategy/openfoodtox> | D |

| **e-resource**  **name** | **Description** | **Potential application in qAOP** | **e-tool reference/link** | **Type of**  **e-resources** |
| --- | --- | --- | --- | --- |
| OpenRiskNet - Risk assessment e-infrastructure | Databases, literature collections and text mining to predictive tools. Things of specific interest are ToxCast/Tox21 and TG-GATEs/DrugMatrix, where OpenRiskNet provided easier access routes for automation, ToxPlanet, ToxicoDB, SCAIView, WikiPathways, BridgeDb, and ToxTargetLinks. | Gaining information on chemicals and biology (input data) | <https://openrisknet.org/e-infrastructure/services/> | D |
| OptiTox | Underdevelopment update of the OpenFoodTox database | Information on chemical hazard data (input data) | <https://www.vegahub.eu/optitox-project-by-efsa/> | D |
| PUBChem | PubChem is an open chemistry database at the National Institutes of Health (NIH). “Open” means that you can put your scientific data in PubChem and that others may use it. PubChem mostly contains small molecules, but also larger molecules such as nucleotides, carbohydrates, lipids, peptides, and chemically-modified macromolecules. We collect information on chemical structures, identifiers, chemical and physical properties, biological activities, patents, health, safety, toxicity data, and many others. | Gaining information on chemicals (input data) | <https://pubchem.ncbi.nlm.nih.gov/> | D |
| Pubmed, Web of Science | Access to the fundamental scientific basis of mechanistic toxicology | Gaining Mechanistic Knowledge (input data, code development information) | <https://apps.webofknowledge.com/>  <https://pubmed.ncbi.nlm.nih.gov/> | K |
| R/Python, Jupyter Notebook | programming languages | Tools for modelling: Can be applied to develop models and qAOP. By targeting each individual KE , KER but also linking them together. (Model code) | https://www.r-project.org/ - https://www.python.org/ - https://jupyter.org/ | M |

| **e-resource**  **name** | **Description** | **Potential application in qAOP** | **e-tool reference/link** | **Type of**  **e-resources** |
| --- | --- | --- | --- | --- |
| Review by Madden et al (2009) | Comprehensive collation of predictive tools and databases to assist development and evaluation of PBK models. Identification of data sources for existing PBK models and overview of bespoke PBK modelling software. Review of efforts to harmonise PBK model construction, evaluation and reporting to increase uptake and acceptance of models. | Exhaustive list of available e-resource for PBK model development and application, that can be used for qAOP | [4] | K |
| sysrev | platform for collaborative extraction of data from academic articles and abstracts, PDF documents, and other entities. | Gaining information on chemicals and biology (input data) | <https://sysrev.com/> | D |
| Text mining tools | Extract the knowledge from different sources (like data, modelling information, Mode of Actions – biological and pathobiological processes etc.) | Collect knowledge | e.g Abstract Sifter[5] (see below), Swift review [6], DistillerSR [7] | K |
| The US EPA tools Abstract Shifter and Chemical lists | The Abstract Sifter is a Microsoft Excel based application that enhances the user experience associated with literature searching of PubMed. | Tools for data mining (Knowledge and data extraction, for qAOp development and execution) | [https://cfpub.epa.gov/si/si_public_record_report.cfm?Lab=NCCT&dirEntryId=341820 - https://www.epa.gov/epcra/consolidated-list-lists](https://cfpub.epa.gov/si/si_public_record_report.cfm?Lab=NCCT&dirEntryId=341820) | AT -D |
| ToxPi: Toxicological Prioritization Index | The ToxPi interface is distributed as a single compressed .zip download. It includes the latest version of the main Java executable, all libraries, example data files, and a complete user manual. This distribution is free, under the GNU GPL. | Dynamic exploration, visualization, and sharing of integrated data models | [https://toxpi.org/](https://urldefense.com/v3/__https:/toxpi.org/__;!!DOxrgLBm!W-4v4r1to4MHCxyE_NSJrmGS-SOBAP9yV2-M1miy4NiZyUpet7QawOuWql4aR40iZpYkzA$) | AT (Visualisation) |

| **e-resource**  **name** | **Description** | **Potential application in qAOP** | **e-tool reference/link** | **Type of**  **e-resources** |
| --- | --- | --- | --- | --- |
| TOXRefDB | ToxRefDB provides detailed chemical toxicity data in a publically accessible searchable format. ToxRefDB contains mammal toxicity information that when combined with other sources of information, such as exposure and metabolism, form the basis for pesticide risk assessments. | Gaining information on chemicals (input data) | <https://cfpub.epa.gov/si/si_public_record_report.cfm?Lab=NCCT&dirEntryId=227139> | D |
| US EPA –CompTox Chemicals Dashboard | The Dashboard is a one-stop-shop for chemistry, toxicity and exposure information for over 875,000 chemicals. Data and models within the Dashboard also help with efforts to identify chemicals of most need of further testing and reducing the use of animals in chemical testing. | Gaining information on chemicals (input data) | <https://comptox.epa.gov/dashboard> | D |
| Web of Science | Citation database, research engine retrieval of best-in-class publication | Gaining Mechanistic Knowledge (input data, code development information) | <https://apps.webofknowledge.com/> | K-D |
| WekaBayes net (also available as KNIME extension) | Software to learn a Bayesian network. | May be used to train a Bayesian model based on a training data set. | <https://www.cs.waikato.ac.nz/~remco/weka.bn.pdf>  <https://www.knime.com/> | M |

**References**

[1] M.E. Pittman, S.W. Edwards, C. Ives, H.M. Mortensen, AOP-DB: A database resource for the exploration of Adverse Outcome Pathways through integrated association networks, Toxicol. Appl. Pharmacol. 343 (2018) 71–83. https://doi.org/10.1016/j.taap.2018.02.006.

[2] M. Martens, C.T. Evelo, E.L. Willighagen, Providing Adverse Outcome Pathways from the AOP-Wiki in Semantic Web Format to Increase Usability and Accessibility of the Content, ChemRxiv. Prepr. (2021). https://doi.org/10.26434/chemrxiv.13524191.v1.

[3] H. van der Voet, J.W. Kruisselbrink, W.J. de Boer, M.S. van Lenthe, J.J.B. (Hans) van den Heuvel, A. Crépet, M.C. Kennedy, J. Zilliacus, A. Beronius, C. Tebby, C. Brochot, C. Luckert, A. Lampen, E. Rorije, C. Sprong, J.D. van Klaveren, The MCRA toolbox of models and data to support chemical mixture risk assessment, Food Chem. Toxicol. 138 (2020) 111185. https://doi.org/https://doi.org/10.1016/j.fct.2020.111185.

[4] J.C. Madden, G. Pawar, M.T.D. Cronin, S. Webb, Y.-M. Tan, A. Paini, In silico resources to assist in the development and evaluation of physiologically-based kinetic models, Comput. Toxicol. 11 (2019) 33–49. https://doi.org/https://doi.org/10.1016/j.comtox.2019.03.001.

[5] N. Baker, T. Knudsen, A. Williams, Abstract Sifter: a comprehensive front-end system to PubMed., F1000Research. 6 (2017). https://doi.org/10.12688/f1000research.12865.1.

[6] SWIFT-Review, Sciome Workbench for Interactive computer-Facilitated Text-mining- Review. https://www.sciome.com/swift-review/ (accessed April 22, 2021).

[7] DistillerSR. https://www.evidencepartners.com/products/distillersr-systematic-review-software (accessed April 22, 2021).
